# A moderate dosage of coffee causes acute retinal capillary perfusion decrease in healthy young individuals

**DOI:** 10.1186/s12886-022-02638-x

**Published:** 2022-11-30

**Authors:** Xiaofeng Zhu, Jiong Zhu, Yongyi Wang, Zhongdi Chu, Ruikang K. Wang, Yi Xu, Lina Lu, Haidong Zou

**Affiliations:** 1 Department of Preventive Ophthalmology, Shanghai Eye Disease Prevention and Treatment Center, Shanghai Eye Hospital, No. 380 Kangding Road, 200040 Shanghai, China; 2 Department of Ophthalmology, Shanghai General Hospital, Shanghai Jiao Tong University, No. 100 Haining Road, 200080 Shanghai, China; 3 Shanghai Key Laboratory of Ocular Fundus Diseases, Shanghai General Hospital, Shanghai Jiao Tong University, Shanghai, China; 4 Shanghai Engineering Center for Visual Science and Photomedicine, Shanghai General Hospital, Shanghai Jiao Tong University, Shanghai, China; 5 Department of Radiology, Renji Hospital, Shanghai Jiao Tong University School of Medicine, Shanghai, China; 6 Department of Medical Administration, Huizhou First Hospital, Guangdong, China; 7 Departments of Bioengineering and Ophthalmology, University of Washington, Seattle, WA USA

**Keywords:** Retinal microcirculation, Retinal capillary perfusion, Caffeine consumption, Spectral-domain optical coherence tomography angiography, Magnetic resonance imaging

## Abstract

**Aim:**

Caffeinated beverages are very popular across populations and cultures, but quantitative evidence of the acute effects of moderate coffee doses on retinal perfusion is sparse and contradicting. Thus, the aim of this randomized, cross-over and parallel-group design study was to investigate whether moderate consumption of coffee alters macular retinal capillary perfusion in young healthy individuals.

**Methods:**

Twenty-seven young healthy individuals were recruited for this study. Acute changes in retinal microvasculature were assessed using spectral-domain optical coherence tomography angiography (SD-OCTA) at baseline, 0.5 h, and 2 h after intake of coffee, or water. Meanwhile, cerebral blood flow (CBF) and retina-choroid blood flow were evaluated in a parallel-group design (4 participants each in coffee or water group) using magnetic resonance imaging (MRI) with pseudo-continuous arterial spin labeling sequences.

**Results:**

Two hours after coffee intake, blood caffeine concentration increased from 0 to 5.05 ± 1.36 µg/mL. Coffee caused a significant decrease in retinal vessel diameter index (VDI) (19.05 ± 0.24 versus [vs] 19.13 ± 0.26; p < 0.001) and CBF in the frontal lobe (77.47 ± 15.21 mL/100 mL/min vs. 84.13 ± 15.55 mL/100 mL/min; p < 0.05) 2 h after intake. However, it significantly increased retina-choroid blood flow after 0.5 and 2 h (163.18 ± 61.07 mL/100 mL/min vs. 132.68 ± 70.47 mL/100 mL/min, p < 0.001, and 161.21 ± 47.95 vs. 132.68 ± 70.47; p < 0.001, respectively).

**Conclusion:**

This is the first study to demonstrate the acute effects of daily dose coffee consumption on retinal capillary perfusion using SD-OCTA combinate with blood flow MRI. The findings imply that although moderate coffee intake caused a significant increase in retina-choroid blood flow, there was a significant acute decrease both in macular retinal capillary perfusion and CBF.

## Background

There is growing awareness that daily consumption of foods, including beverages, can provide a preventive approach to improved health. Caffeinated beverages are among the most habitually consumed foods across populations and cultures [1]. Caffeine is a recognized risk factor for hypertension, although prospective and epidemiological studies have not demonstrated a positive correlation between habitual coffee consumption and cardiovascular disease [2, 3].

Although recent studies have demonstrated a significant decrease in fingertip and macular blood flow after caffeine intake [4, 5], others argue that caffeine causes an increase in endothelium-dependent vasodilatation [6, 7]. Nevertheless, little is known about the acute effects of a moderate dosage of coffee on retinal microvasculature or blood flow. Changes in retinal microvasculature have been demonstrated to be significantly correlated with the occurrence and development of ophthalmic diseases, and closely related to systemic diseases (e.g., diabetic retinopathy and retinal vein obstruction) [8, 9]. Therefore, the evaluation of the acute effects on retinal microvasculature in healthy and diseased individuals remains worthwhile.

Recently, the development of spectral-domain optical coherence tomography angiography (SD-OCTA) has enabled direct visualization and quantitative analysis of the retinal and choroidal vasculature in a quick and noninvasive manner [10, 11]. Meanwhile, magnetic resonance imaging (MRI) can quantitatively evaluate cerebral blood flow (CBF) and retina-choroid blood flow using the noninvasive arterial spin labeling (ASL) technique in a large field of view (FOV) without depth limitations [12, 13]. Therefore, the aim of this study was to investigate whether moderate dosage of coffee consumption changed cerebral and macular retinal capillary perfusion over a given period of time based on the peak concentration of caffeine in healthy young adults using SD-OCTA and MRI.

## Methods

### Participants

Young university students were recruited through public advertisements in the Shanghai Eye Disease Prevention and Treatment Center from March to June 2019. The inclusion criteria were: (1) aged 18–30 years; (2) with no history of ocular, systemic, or neurologic disease; (3) non-smokers (never smoke), non-drinkers (never/almost never drink), and not heavy coffee drinkers (<2.5 cups/day) [14, 15]; and (4) without refractive errors (spherical equivalent degree ≤ -3 diopters). Participants were asked to refrain from eating and drinking any caffeine- or alcohol-containing products for at least 24 h before the experiment, avoid strenuous exercise on the day of the experiment, and have > 8 h of sleep the night before the experiments. Moreover, female participants were asked to avoid participating in the experiment during their menstrual period.

This study was approved by the Institutional Review Boards of Shanghai General Hospital and adhered to the tenets of the Declaration of Helsinki. Written informed consent was obtained from all study participants before enrollment. All participants were compensated for participation in this study.

### Protocol

All participants underwent a comprehensive ocular examination, including autorefraction and visual acuity assessment, intraocular pressure (IOP), and axial length measurement, before the experiments, and reported frequency of total coffee intake per day in their daily life. This was a randomized cross-over and parallel-group design trial in which participants were required to complete two experimental sessions and consume either coffee or water alone. Participants visited the research laboratory on two mornings separated by a one-week washout period. If the participant randomly drinks coffee on the first visit, he or she will be asked to drink water in the second test, and vice versa. On arrival, the participants ate a standardized low-calorie meal, and were subsequently instructed not to eat for 2 h before baseline scanning [6]. Then, participants drank 300 ml of coffee containing 72 mg of caffeine per 6 g ground coffee without sugar or milk (instant coffee, UCC Ueshima Coffee Co., Ltd., Tokyo, Japan), or water, respectively. Whether the participants accept the MRI scan or not was on a voluntary basis, and they were randomly divided into two groups (coffee and water) to undergo three MRI scans. SD-OCTA, MRI, blood pressure (BP), heart rate (HR), and IOP were measured at baseline, 0.5 and 2 h after coffee consumption, based on the immediate effect (0.5 h) and peak concentration of caffeine (2 h) absorption through the gastrointestinal tract [16]. Venous blood was collected before each scan. Blood glucose and plasma levels of caffeine were measured using liquid chromatography.

### Equipment

SD-OCTA (Cirrus, Carl Zeiss Meditec, Inc., Dublin, CA, USA) images were acquired using a Cirrus prototype with a central wavelength of 840 nm and a scan speed of 68,000 A-scans per second. Participants were instructed to focus on a fixation target while a 3 × 3 mm fovea-centered scan of the right eye was acquired and repeated three times, with signal strength above 6 and avoidance of significant artifactual components (e.g., floaters, eyelashes, motion, or other artifacts) [17]. A complex intensity-based optical microangiography algorithm was used to generate full-thickness en face OCTA images. An automated segmentation algorithm produced three horizontal depth-resolved slabs consisting of the superficial retinal layer (SRL), encompassing the superficial 60% of retina extending from the inner limiting membrane (ILM) to 110 μm above the retinal pigment epithelium (RPE), deep retinal layer (DRL), extending through the remaining 40% of the thickness between the ILM and RPE, and overall retinal layer (ORL), extending from the ILM to the RPE [10, 11]. The images are processed into a binary image in MATLAB (R2015b, MathWorks, Inc.) using a global threshold, hessian filter, and adaptive threshold. The global threshold is determined by the noise level in the foveal avascular zone (FAZ) that is selected with a user interactive interface by asking the operator to click on the center and edge of FAZ. This global threshold is then applied to the entire image. Then, hessian filter and adaptive threshold are combined to generate a binary vessel map to describe the retinal microvascular density and morphology. These parameters included vessel diameter index (VDI), vessel area density (VAD), vessel skeleton density (VSD), and flow impairment region (FIR), and all of the binarization algorithms show good reliability and repeatability in quantitative assessment of the retinal microvascular changes using SD-OCTA [10, 11].

MRI studies were performed using a 3.0 Tesla whole body clinical scanner (DiscoveryTM MR750, GE Healthcare, Milwaukee, WI, USA) equipped with an eight-channel array head coil and a custom-built single-loop radiofrequency coil (round shape, 47-mm in diameter) mounted on a bracket and fixed in front of the right eye. CBF and retina-choroid blood flow were imaged using the pseudo-continuous ASL sequence combining fast spin-echo imaging with background suppression to achieve whole-brain and single-eye coverage. Scanning sequences for CBF were as follows: repetition time (TR), 4563 ms; echo time (TE), 9.69 ms; voxel size, 4 × 1.7 × 1.7 mm^3^; FOV, 24 mm; flip angle, 180°; post label delay time, 1525 ms; number of excitations (NEX), 6; and receiver bandwidth, ± 62.50 kHz [12]. To investigate retina-choroid blood flow, a single axial slice, roughly bisecting the optic nerve head (ONH), was imaged, and participants were instructed to blink to minimize eye motion during MRI scanning. The acquisition parameters were as follows: TR, 4563 ms; TE, 9.69 ms; voxel size, 4 × 1.7 × 1.7 mm^3^; FOV, 18 mm; flip angle, 180°; post label delay time, 1525 ms; NEX, 6; and receiver bandwidth, ± 62.50 kHz [18]. Image evaluation was performed on the FUNCTOOL workstation (AW4.2, GE Healthcare). The frontal lobe was selected as the region of interest (ROI) to measure and average the change in CBF [12]. Blood flow for the entire retinal thickness was determined as a function of the distance from the ONH, where the blood flow was measured at the peaks of the projection profiles. An ROI outlining the posterior retina with a size of 0.7 to 0.8 mm across the retina-choroid thickness in the parafoveal region was used to obtain the blood flow signal time courses and averaged values [13, 18].

### Data analysis

Statistical analysis was performed using SPSS version 21.0 (IBM Corporation, Armonk, NY, USA), and P < 0.05 was considered statistically significant. Given that no literature containing the standard deviation parameter of retinal capillary perfusion, the study was designed in an exploratory basis with 90% power and two-sided 5% significance. 25 of the sample size per group for small (0.2) standardized effect sizes was recommended [19]. The Kolmogorov–Smirnov test was used to examine distribution of the main quantitative variables. The chi-squared test was used to examine the relationship between dichotomous variables in two groups. Baseline SD-OCTA and MRI measurements were compared using one-way analysis of variance (ANOVA) in two groups. In addition, repeated measures ANOVA was used to compare the SD-OCTA and MRI measurements in three time points.

## Results

Twenty-seven university students (12 males, 15 females), with a mean (± standard deviation [SD]) age of 22.7 (± 3.1) years, mean weight of 60.4 (± 15.0) kg, mean height of 165.2 (± 6.9) cm, and mean body mass index of 22.0 (± 4.5) kg/m^2^, participated in the study. Participants’ best-corrected visual acuity in the worse eye were all ≥ 20/20, with a mean spherical equivalent of -1.1 (± 1.5) diopters, mean IOP of 14.6 (± 2.7) mmHg, and mean axial length of 23.9 (± 1.0) mm. The mean daily cups of coffee consumption was 0.96(± 0.71), with a minimum of 0 and a maximum of 2. Nineteen participants dropped out of the MRI scan for a variety of reasons (e.g., fear of MRI examination, not available for the scheduled time), and 8 participants using a parallel-group design were included and randomly divided into two groups (coffee and water), with two males and two females in each group.

### Systemic and blood parameters

Two hours after drinking coffee, plasma caffeine levels were significantly higher compared to 0.5 h after drinking. There were no significant differences in systolic or diastolic BP, HR, IOP, or blood glucose at baseline. Although ANOVA depicted no significant interaction among HR, IOP, or blood glucose, systolic and diastolic BP increased significantly 2 h after drinking coffee compared to baseline (Table 1).

**Table 1:** Systemic and blood parameters before and after drinking coffee or water (n = 27)

	Coffee	Water
**Baseline**		
P-caffeine (µg/mL)	-	-
Blood glucose (mmol/L)	4.81 ± 0.46	5.15 ± 0.43
Systolic blood pressure (mm Hg)	114.18 ± 11.33	118.00 ± 14.32
Diastolic blood pressure (mm Hg)	69.36 ± 6.82	73.75 ± 8.07
Heart rate (bpm)	74.77 ± 12.43	72.88 ± 2.85
Intraocular pressure (mmHg)	14.5 ± 1.7	14.6 ± 2.1
**After drinking**		
P-caffeine (µg/mL)		
0.5 h	3.09 ± 1.64	-
2 h	5.05 ± 1.36	-
Blood glucose (mmol/L)		
0.5 h	5.35 ± 0.42	5.25 ± 0.39
2 h	6.09 ± 0.39	4.98 ± 0.27
Systolic blood pressure (mm Hg)		
0.5 h	118.68 ± 12.73	118.75 ± 12.00
2 h	120.18 ± 15.31^*^	117.75 ± 13.12
Diastolic blood pressure (mm Hg)		
0.5 h	70.77 ± 8.35	73.88 ± 5.25
2 h	74.59 ± 11.45^*^	74.00 ± 8.54
Heart rate (bpm)		
0.5 h	72.05 ± 12.82	68.63 ± 6.28
2 h	74.55 ± 18.58	69.38 ± 3.46
Intraocular pressure (mmHg)		
0.5 h	15.1 ± 1.5	15.1 ± 1.9
2 h	14.7 ± 2.4	15.8 ± 2.0

Values are mean ± SD. -, not detected; bmp, beats per minute

Reference group: Baseline. Repeated measures analysis of variance, significance level: *p < 0.05

### Retinal capillary perfusion

**Fig. 1:**
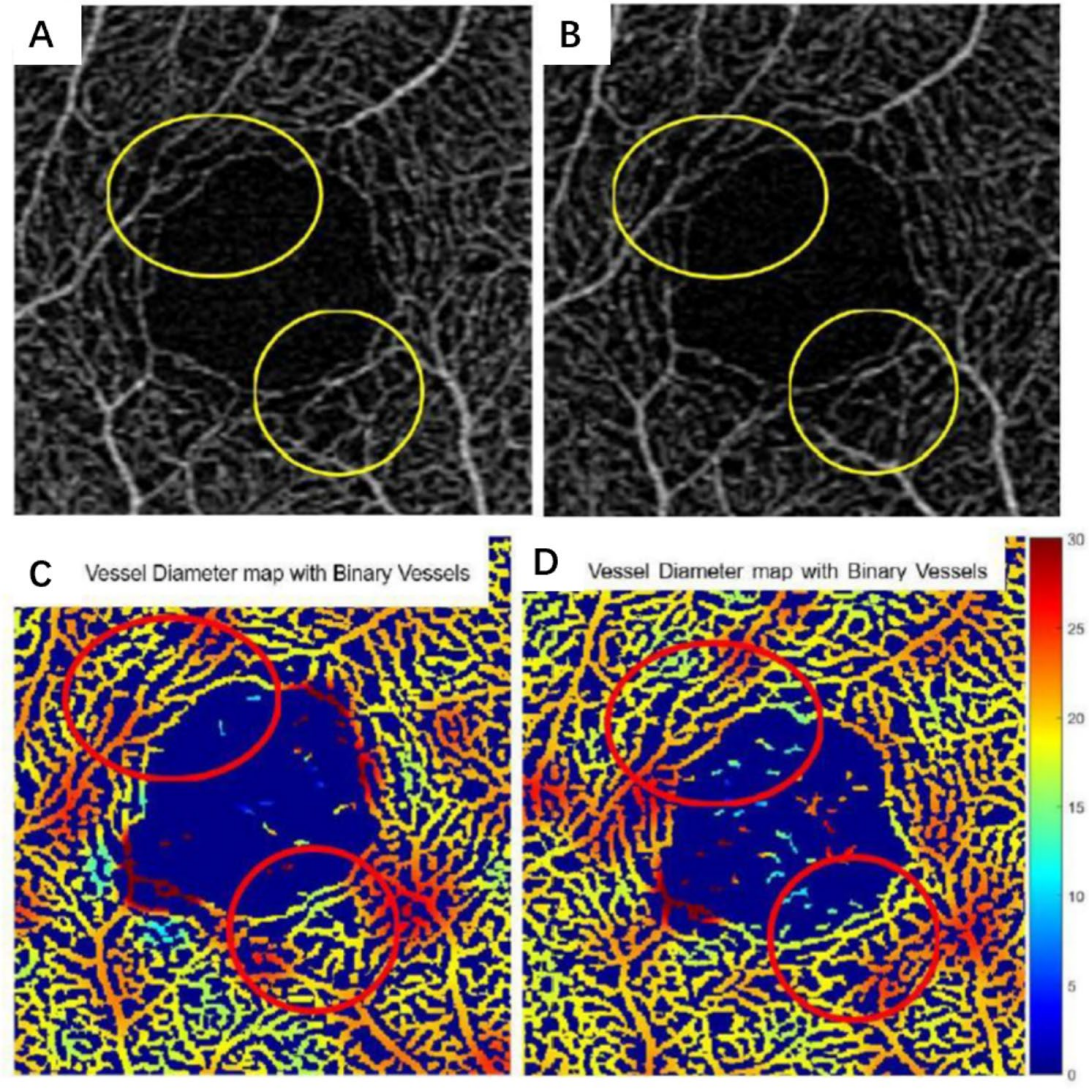
Superficial retinal layer of SD-OCTA images in 3 × 3-mm areas around the fovea in healthy individuals. (A, B): original *en* face OMAG image. (C, D): vessel diameter map with binary vessels (inµm). (A-D): Representative eye from a healthy 23-year-old female subject. The yellow circles in (A) and (B) show visible macular arch ring shrink and break, and flow impairment region enlargement before and after drinking coffee. (C, D): red circles show that regions in dark red demonstrated a significant decrease in the superficial retinal layer from baseline to 2 h after drinking coffee

A total of twenty-seven participants underwent the SD-OCTA measurement. The signal strength of all images included in the study were all higher than or equal to 8. Two eyes (0 for baseline, 1 for 0.5 h, and 1 for 2 h) were excluded from the study because of significant artifacts with at least one of the three scans. There were no significant differences in VDI, VAD, VSD or FIR in the SRL, DRL, or ORL at baseline among the participants before drinking coffee or water (Table 2). Compared to baseline, drinking coffee caused a significant decrease in VDI after 2 h, both in the SRL and ORL, while there was no significant difference in the DRL (Fig. 1). None of VAD, VSD or FIR were significantly changed in the SRL, DRL, or ORL after drinking coffee when compared to baseline.

**Table 2:** Macular perfusion parameters before and after drinking coffee or water

		Baseline (n = 27)	0.5 h after drinking (n = 26)^#^	2 h after drinking (n = 26)^#^
**Coffee**				
Superficial retinal layer	Vessel diameter index	19.13 ± 0.26	19.11 ± 0.25	19.05 ± 0.24^‡^
	Vessel area density	0.47 ± 0.01	0.46 ± 0.01	0.47 ± 0.02
	Vessel skeleton density	0.14 ± 0.00	0.14 ± 0.00	0.14 ± 0.01
	Flow impairment region^a^	0.38 ± 0.18	0.39 ± 0.15	0.45 ± 0.26
Deep retinal layer	Vessel diameter index	18.43 ± 0.19	18.37 ± 0.20	18.36 ± 0.22
	Vessel area density	0.45 ± 0.02	0.45 ± 0.02	0.45 ± 0.02
	Vessel skeleton density	0.14 ± 0.00	0.14 ± 0.00	0.14 ± 0.01
	Flow impairment region	0.33 ± 0.11	0.34 ± 0.16	0.32 ± 0.12
Overall retinal layer	Vessel diameter index	18.73 ± 0.23	18.70 ± 0.24	18.66 ± 0.22^†^
	Vessel area density	0.48 ± 0.01	0.48 ± 0.01	0.48 ± 0.02
	Vessel skeleton density	0.15 ± 0.00	0.15 ± 0.01	0.15 ± 0.01
	Flow impairment region	0.35 ± 0.13	0.36 ± 0.13	0.38 ± 0.20
**Water**				
Superficial retinal layer	Vessel diameter index	19.08 ± 0.29	19.10 ± 0.30	19.08 ± 0.27
	Vessel area density	0.47 ± 0.01	0.47 ± 0.01	0.47 ± 0.01
	Vessel skeleton density	0.14 ± 0.00	0.14 ± 0.00	0.14 ± 0.00
	Flow impairment region	0.35 ± 0.17	0.36 ± 0.19	0.35 ± 0.17
Deep retinal layer	Vessel diameter index	18.42 ± 0.24	18.39 ± 0.24	18.41 ± 0.23
	Vessel area density	0.46 ± 0.02	0.46 ± 0.01	0.46 ± 0.02
	Vessel skeleton density	0.14 ± 0.00	0.14 ± 0.00	0.14 ± 0.00
	Flow impairment region	0.28 ± 0.12	0.27 ± 0.10	0.29 ± 0.14
Overall retinal layer	Vessel diameter index	18.68 ± 0.24	18.70 ± 0.27	18.67 ± 0.24
	Vessel area density	0.49 ± 0.01	0.48 ± 0.01	0.49 ± 0.01
	Vessel skeleton density	0.15 ± 0.00	0.15 ± 0.00	0.15 ± 0.00
	Flow impairment region	0.33 ± 0.15	0.33 ± 0.11	0.33 ± 0.13

Values are mean ± SD.

^a^, Flow impairment region is measured in square millimeters (mm^2^)

Reference group: Baseline. Repeated measures analysis of variance, significance level: *p < 0.05; †p < 0.01; ‡p < 0.001

^#^, Images of one participant were excluded from analysis due to artifacts

### Retina-choroid blood flow

**Fig. 2:**
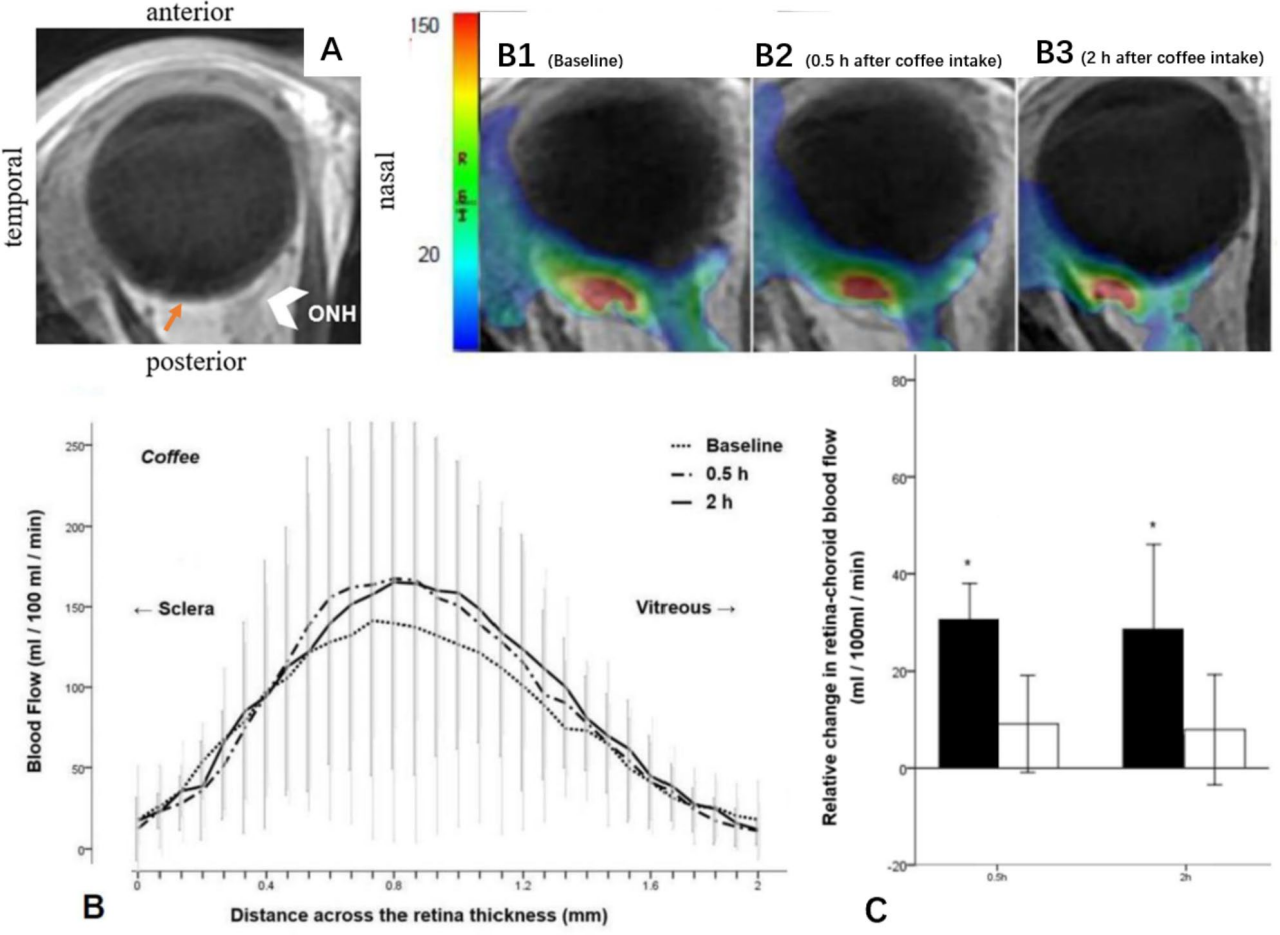
Blood flow across retina-choroid thickness compared to baseline after drinking coffee, as imaged with pCASL-MRI. (A) Scout image and slice position. Orange thin arrowhead roughly indicates the foveal region, and white thick arrowhead indicates the optic nerve head (ONH). (B) Blood flow profile before and after coffee intake across the retina-choroid thickness from the sclera to the vitreous. (B1-B3): representative quantitative blood flow in rainbow scale with unit of mL/100 mL/min from a healthy 22-year-old male subject. (C) The relative change in retina-choroid blood flow from baseline at 0.5 and 2 h after drinking coffee and water. Values are mean ± SD. *P < 0.05 vs. baseline

Eight participants were performed the MRI scan, four in coffee group and four in water group. There were no significant differences in retina-choroid blood flow at baseline among the participants before drinking coffee or water. Although no statistically significant correlation between blood caffeine concentration and retina-choroid blood flow was found, drinking coffee significantly increased retina-choroid blood flow when compared to baseline (163.18 ± 61.07 vs. 132.68 ± 70.47 mL/100 mL/min, and 161.21 ± 47.95 vs. 132.68 ± 70.47 mL/100 mL/min, respectively, at 0.5 and 2 h after drinking coffee, p < 0.001) (Table 3, and Fig. 2). In addition, there were no significant differences in retina-choroid blood flow after drinking water compared to baseline.

**Table 3:** Cerebral blood flow and retina-choroid blood flow parameters (mL/100 mL/min) before and after drinking coffee (n = 4) or water (n = 4)

	Baseline	0.5 h after drinking	2 h after drinking
**Cerebral blood flow**			
Coffee	84.13 ± 15.55	83.59 ± 11.24	77.47 ± 15.21*
Water	85.60 ± 18.19	85.85 ± 15.05	82.10 ± 18.15
**Retina-choroid blood flow**			
Coffee	132.68 ± 70.47	163.18 ± 61.07^‡^	161.21 ± 47.95^‡^
Water	107.23 ± 37.89	116.36 ± 29.74	115.16 ± 12.77

Values are mean ± SD.

Reference group: Baseline. Repeated measures analysis of variance, significance level: *p < 0.05; †p < 0.01; ‡p < 0.001

### CBF MRI

**Fig. 3:**
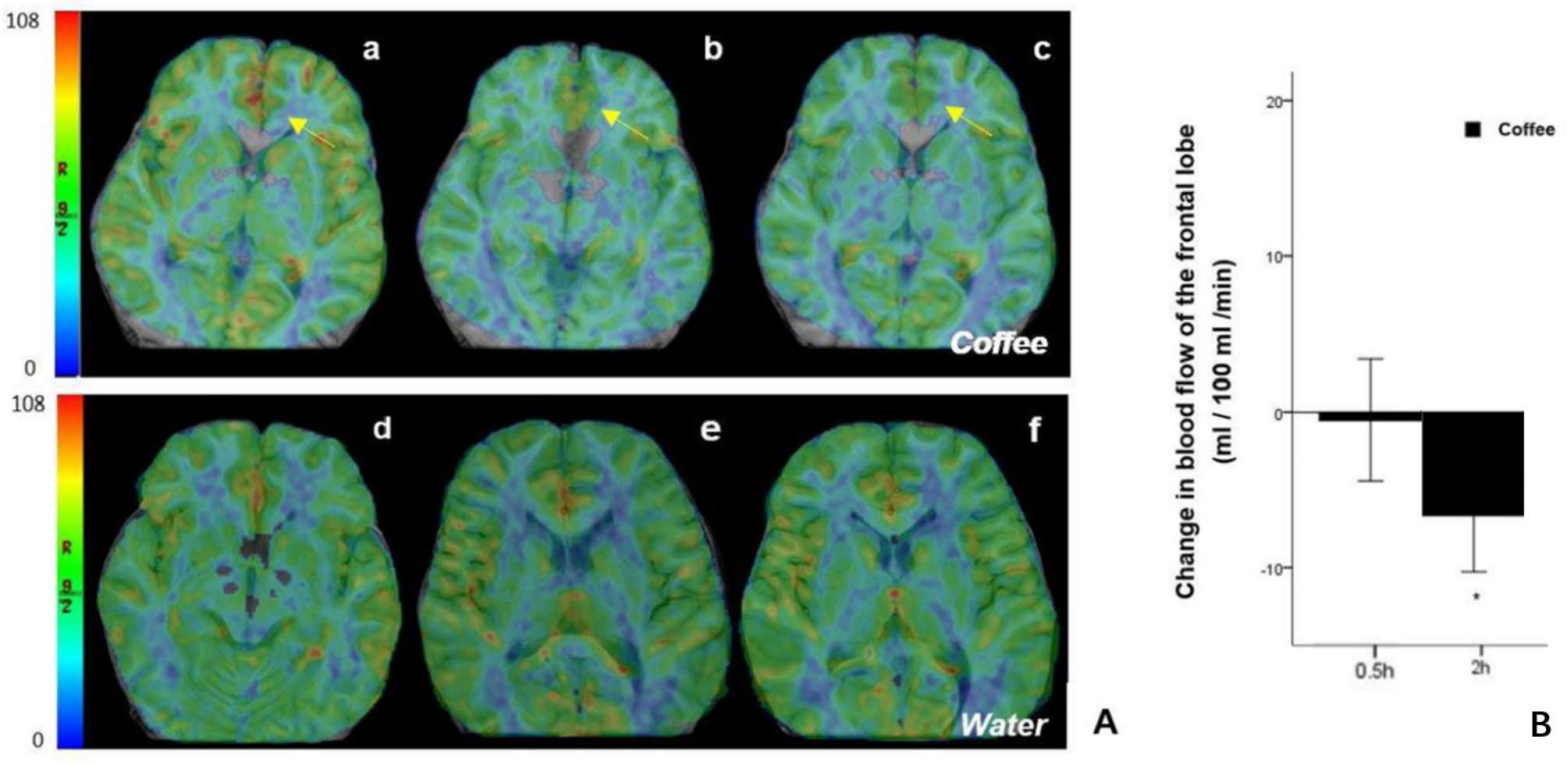
Effect on cerebral blood flow as imaged with pCASL-MRI. (A) Frontal lobe map as the region of interest (ROI) in different groups. (a-c) Baseline, 0.5 h, and 2 h after drinking coffee; (d) - (f) Baseline, 0.5 h, and 2 h after drinking water. The yellow arrows demonstrated a significant change in blood flow in these regions. (B) Change in averaged CBF (mL/100 g/min) value extracted from the circled cluster compared to baseline after drinking coffee and plotted by session and time. The vertical lines represent the standard error of the mean. *P < 0.05 vs. baseline

There were no significant differences in CBF at baseline among the participants before drinking coffee or water. Compared to baseline, CBF in the ROI was significantly decreased 2 h after drinking coffee (77.47 ± 15.21 vs. 84.13 ± 15.55 mL/100 mL/min, p < 0.05) (Table 3, and Fig. 3).

## Discussion

In this randomized cross-over and parallel-group design trial, we demonstrated the acute effects of daily dose coffee consumption on retinal capillary perfusion using SD-OCTA, combinate with retina-choroid blood flow and CBF using blood flow MRI. Although moderate coffee intake caused a significant increase in retina-choroid blood flow detected by MRI scan, there was an acute significant decrease both in retinal capillary perfusion and CBF.

Compared to baseline, we found that even drinking moderate coffee caused a significant decrease in retinal VDI with elevated BP after 2 h, although no significant changes in HR were detected. As we known, caffeine is an adenosine receptor antagonist, and adenosine is one of the most important endogenous vasodilative substances [20]. Similar result was observed in the previous research, retinal vessel diameter acutely decreased and was negatively correlated with mean systemic arterial pressure after 200 mg oral caffeine intake in young healthy participants [21], which appeared to be elicited by an autoregulatory response. However, none of retinal VAD, VSD or FIR were significantly changed after coffee consumption in young healthy participants in our study, while VDI changed. VDI presents the vessel size information regardless of the vessel length (VSD); therefore, it is sensitive to vascular dilation/constriction in the OCTA images. VAD provides the best estimate of real vessel density as it takes both vessel length and vessel diameter into consideration. However, a complication would be that VAD remains unchanged when there are decreased perfusion and vessel dilation happening at the same time, potentially resulting in false negatives on the vascular abnormality [10, 11].

There was a measurable reduction in CBF (the frontal lobe as ROI) after drinking a moderate dosage of coffee, which is consistent with the results of retinal capillary perfusion in our and a previous study [22]. In previous studies, caffeine has been found significantly affected the frontal BOLD fMRI signal [23, 24]. Although widespread effects of acute caffeine on cerebral perfusion also demonstrated regional, dose-dependent, and inter-individual variation, the frontal lobe was selected as the ROI to measure and average the change in CBF after coffee consumption. The finding that these frontal regions appear to be sensitive to the vasoactive effects of caffeine, which is consistent with our present study.

In contrast to a decrease in retinal capillary perfusion, retina-choroid blood flow significantly increased after coffee consumption compared to baseline when measured by MRI. However, retina-choroid blood flow slightly increased after water consumption, probably because of volume effect, it was no statistically significant from baseline. In previous studies, ophthalmic blood vessel resistance increased and blood flow in human ONH and choroid-retina decreased after caffeine intake, when measured by laser speckle analyzer or color doppler ultrasonography, which was inconsistent with our findings [25, 26]. In addition, more recent studies have demonstrated that caffeine intake caused a significant decrease in choroidal thickness by spectral-domain OCT, which may be related to reduced ocular blood flow due to its vasoconstrictive effect [27, 28]. However, there was no significant change observed in ocular pulse amplitude, and sub-foveal choroidal thickness was not found to significantly correlate with choroidal blood flow in young healthy eyes after caffeine intake [29, 30].

Owing to the complex nature of the ocular posterior pole, which is nourished by two vascular beds (the retina and choroid), it is conceivable that one measurement tool may not be sufficient to accurately assess the retinal vessels and blood flow [31]. Therefore, two measurement methods were used to quantify and analyze ocular blood flow from different planes. In addition to the ocular microvasculature, several studies have found contradicting acute effects of caffeine consumption in various vasculature of the human body. Most studies have reported a slight increase in BP and arterial stiffness, decreased glucose disposal, and impaired endothelial function [22, 32–34]. But some studies have found that caffeine caused an increase in endothelium-dependent vasodilatation [6, 7]. Possible explanations are that caffeine acted as a competitive inhibitor of A1, A2a, and b-adenosine receptors, and increased nitric oxide production, to shift the balance between vasodilatory and contractile mechanisms [6, 20]. Moreover, different sources of retina-choroid blood supply, different distribution of adenosine receptors, and retinal autoregulatory response may explain these conflicting findings [35]. Therefore, further prospective studies are required to determine whether choroidal thickness is positively correlated with choroidal blow flow, and the precise underlying mechanisms of caffeine on ocular microvasculature needs to be explored more deeply.

It should be noted that the caffeine dosage used in our study was a typical daily dosage, which is much lower than that used in other studies. Nevertheless, it caused a measurable change in retinal capillary perfusion, retina-choroid blood flow, and CBF in healthy participants. Compared to younger and healthier people, caffeine may elevate BP and constrict retinal vessels more effectively in older individuals and those with hypertension, diabetes, or retinal microvascular disorders [36, 37]. Therefore, it reminds us the possible health risks after drinking a moderate dosage of coffee.

### Strengths and limitations

To our knowledge, our study is the first to demonstrate the acute effects of daily dose coffee on the retinal capillary perfusion using SD-OCTA combinate with blood flow MRI among young healthy participants. These two techniques not only have the advantages of being noninvasive, precise, and repeatable, but also can be combined and verified with each other to find out the actual effect of coffee on ocular blood flow perfusion. We chose drinking coffee through the gastrointestinal tract instead of pure caffeine, and a moderate dosage instead of a high dosage, because it more closely resembles typical daily consumption and eliminated the influence of many confounding variables by selecting young healthy participants. All experiments were performed in the same participants, who also served as an internal control group in the experimental protocol. Furthermore, we monitored changes in blood caffeine concentrations and related metabolic indicators.

The current study has some potential limitations that should be addressed. Firstly, the sample size and the number of time points examined may have been too small to draw definitive correlation between moderate coffee consumption and cerebral and macular retinal capillary perfusion, and large difference between number of participants included in MRI and SD-OCTA measurements was existed in this exploratory study. Secondly, blinding of these participants seems to be unlikely, because the characteristics of coffee and water are obviously different. Several clinical trials comparing the different effects of coffee and water consumption on the human body have not used blinding [38, 39]. However, we agreed that “un-blinded” might have influence on the results due to psychological effect to some extent. In addition, SD-OCTA images represent retinal perfusion and flow/velocity, not anatomical angiograms. Therefore, it is strictly spoken, not possible to assign a change in the angiograms to either a diameter or a velocity change of the vessels, and these parameters will alter due to image quality, repeated measurements, and segmentation errors. Moreover, retinal and choroidal blood flow cannot be differentiated when only measured by MRI, therefore, we combined the detection of SD-OCTA to focus on the changes of the macular retinal capillary perfusion. We are unable to know whether more detectable microvascular responses exist in individuals with retinal microvascular abnormalities or systemic diseases. Furthermore, although baseline values had no statistically difference before drinking coffee or water, high baseline variation remained. Possible reasons for this variation included that in this randomized cross-over and parallel-group design trial, despite these participants were ordered to follow a healthy daily routine before the experiment, they completed two SD-OCTA experimental sessions on two different mornings, and only 8 individuals included in MRI scan and randomly divided into coffee and water groups. Future prospective and interventional studies in large-scale human who are healthy or have underlying medical conditions are warranted to confirm our findings.

## Conclusion

Our study found that, although moderate coffee intake caused a significant increase in retina-choroid blood flow in MRI scan, there was a significant acute decrease both in macular retinal capillary perfusion and CBF. More research is needed to confirm our results and to develop health reference for different individuals.

## Data Availability

The datasets used and analyzed during the current study are available from the corresponding author on reasonable request.

## Abbreviations

SD-OCTA: spectral-domain optical coherence tomography angiography
MRI: magnetic resonance imaging
CBF: cerebral blood flow
ASL: arterial spin labeling
FOV: field of view
SD: standard deviation
IOP: intraocular pressure
BP: blood pressure
HR: heart rate
SRL: superficial retinal layer
DRL: deep retinal layer
ORL: overall retinal layer
VDI: vessel diameter index
VSD: vessel skeleton density
VAD: vessel area density
FIR: flow impairment region
TR: repetition time
TE: echo time
NEX: number of excitations
ONH: optic nerve head
ROI: region of interest
ANOVA: analysis of variance.
